# Pancreatic Pseudocysts with Progressive Multiorgan Dysfunction

**DOI:** 10.51894/001c.164414

**Published:** 2026-08-01

**Authors:** Caesar Abbas, Kiran Sharma, Mohammad Munir Zaitoun, Zeeshan Rizvi, David Neff

**Affiliations:** 1 College of Osteopathic Medicine, Michigan State University

## 54

### Introduction

Pancreatic pseudocysts are recognized complications of acute and chronic pancreatitis, which occur in approximately 5 to 16 percent of cases. Chronic alcohol use is a common underlying cause due to recurrent pancreatic inflammation and duct disruption. Most pseudocysts are solitary and resolve with conservative management. However, the presence of multiple giant pseudocysts greater than 10 cm is uncommon and may produce obstructive symptoms requiring intervention. Management strategies have evolved from surgical cystogastrostomy, first described in 1921, to minimally invasive endoscopic approaches. The introduction of endoscopic ultrasound guidance and stent placement expanded treatment options. Current professional guidelines recognize endoscopic ultrasound-guided drainage as the preferred intervention for symptomatic pancreatic pseudocysts.

### Case Description

An adult male with chronic alcohol use presented with recurrent hospitalizations for pancreatitis complicated by ascites ranging from 6 to 8 L. Magnetic resonance imaging demonstrated two pancreatic pseudocysts measuring approximately 10 to 12 cm, both being in the pancreatic head. One communicated with the pancreatic duct and compressed the liver, while the other compressed the stomach, causing vomiting, gastric outlet obstruction, early jaundice, and pancreatic dysfunction. Management was initially debated, leading to a multidisciplinary conference involving gastroenterology, surgery, and the regional pancreatic tumor board to coordinate care. Due to cyst size and compression, endoscopic ultrasound-guided cystogastrostomy with stent placement was performed to drain both pseudocysts.

### Discussion

Lumen-apposing metal stents (LAMS) were placed to maintain a tract between the pseudocysts and the gastric lumen. These stents maintain tract patency and allow bidirectional fluid movement, although physiologic pressure gradients promote drainage into the stomach. The stents remained in place for 6 to 12 weeks and resulted in the resolution of both pseudocysts with remission of ascites. Concurrent treatment for alcohol use disorders was initiated. This case highlights successful endoscopic management of multiple giant pancreatic pseudocysts causing organ compression. Advances in endoscopic drainage and lumen-apposing metal stents reduced the need for surgical intervention. Multidisciplinary decision making remains essential when severe pancreatic complications arise, and long-term follow-up is necessary as patients with chronic pancreatitis remain at risk for pancreatogenic diabetes.

